# Plot-level impacts of improved lentil varieties in Bangladesh

**DOI:** 10.1371/journal.pone.0262146

**Published:** 2022-01-25

**Authors:** Yigezu Atnafe Yigezu, M. Wakilur Rahman, Tamer El-Shater, Arega D. Alene, Ashutosh Sarker, Shiv Kumar, Aymen Frija

**Affiliations:** 1 International Center for Agricultural Research in the Dry Areas (ICARDA), Cairo, Egypt; 2 Bangladesh Agricultural University (BAU), Mymensingh, Bangladesh; 3 International Center for Agricultural Research in the Dry Areas (ICARDA), Al-Sweida, Syria; 4 International Institute of Tropical Agriculture (IITA), Lilongwe, Malawi; 5 International Center for Agricultural Research in the Dry Areas (ICARDA), New Delhi, India; 6 International Center for Agricultural Research in the Dry Areas (ICARDA), Rabat, Morocco; 7 International Center for Agricultural Research in the Dry Areas (ICARDA), Tunis, Tunisia; Ghazi University, PAKISTAN

## Abstract

The advent of improved lentil varieties (ILVs) in the mid-1990s solved the disease problem which almost halted lentil production in Bangladesh. Levels of adoption of ILVs have been documented in the literature, but little is known about their impacts. Applying an instrumental variables regression to data collected from a sample of 1,694 lentil plots and DNA fingerprinting for varietal identification, this study provides estimates of the plot-level impacts of adoption of ILVs in Bangladesh. Model results show that adoption of ILVs is associated with 14.3% (181.14 kg/ha) higher yields and 17.23% (US$169.44/ha) higher gross margins. Since 45% of lentil area is under ILVs, they generated over 8.77 tones (6%) more supply of lentils from domestic sources, saving the country US$8.22 million in imports in 2015 alone. By investing in the generation and scaling of ILVs, Bangladesh and other South Asian countries with similar agro-ecologies can increase production and decrease dependency on lentil imports.

## Introduction

### Background

Global efforts for documenting the contribution of research to agricultural development, food security, poverty alleviation and other outcomes have mainly focused on major cereal food crops such as wheat, rice, and maize [1–7]. As a result, little attention has been given to the quiet revolution that has taken place due to wide diffusion of improved varieties of food legume crops. Over 99% of lentil area in western Bangladesh is cultivated with short-duration improved lentil varieties (ILVs), which are grown between irrigated rice crops and on unirrigated lands during the dry season [8]. Little is known about the impacts of these varieties. This article documents plot-level impacts of these lentil varieties in Bangladesh where “plot” is defined as a small agricultural land covered by a single type of vegetation. Sometimes, a given agricultural field with a single owner can be divided into two or more plots cultivated with two or more types of vegetation.

Lentil is the most important pulse crop in Bangladesh where it serves as human food, animal feed and fodder, and as a raw material for agro-processing industries [9]. It is rich in protein (25.7–33.4%) and several essential micronutrients [10–12]. Moreover, because it fixes nitrogen, legume production can improve soil fertility [13].

Area under lentils in Bangladesh grew from 62,000 ha in 1961 to 295,000 in 1979. Prior to the 1980s, lentil was grown during the dry season as few alternatives existed to follow the rainy season (*Aman*) rice harvest. In the 1980s, two forces converged, and lentil area declined. First, aggressive expansion of irrigation allowed irrigated dry season (*Boro*) rice to displace lentil production. Second, traditional lentil varieties became vulnerable to two important diseases—Stemphylium blight (*Stemphylium botryosum*) and rust (*Uromyces fabae*) which began to spread in the early 1980s.

In response to the disease problem, the Pulses Research Center (PRC) of Bangladesh Agricultural Research Institute (BARI) and Bangladesh Institute of Nuclear Agriculture (BINA) individually and in collaboration with the International Center for Agricultural Research in the Dry Areas (ICARDA) began breeding disease-resistant lentil varieties. In the mid-1990s, two improved varieties (BARI Masur-3 and BARI Masur-4) with resistance to these diseases were released by BARI. Despite their reduced disease susceptibility, lentils remained at an economic disadvantage to the dry season *Boro* rice, particularly on irrigated lands. Area planted to lentils continued to decline to 90,000 ha in 2013. Meanwhile, BARI and BINA along with ICARDA continued development and release of ILVs which, in addition to disease resistance, are high yielding and more remunerative. As of 2013, eight modern varieties of lentil had been released. The new varieties were also early maturing making it possible to grow them during the short window between rice crops. As a result, area under lentil started to increase reaching 156,000 in 2018—still much lower than 1979 levels but 71% higher than in 2013 [14].

While the biophysical benefits of legumes are well documented, mostly using on-station experimental data, the literature on the socio-economic impacts of improved varieties of legume crops in general and lentils in particular is scant. In a study from two districts in Western Bangladesh [15], fit a Cobb-Douglas production function, estimated net margins of 27,838 (US$339.62) per ha and concludes that adoption of ILVs is profitable. Another study [16] also show that adoption of improved faba-bean varieties in Morocco leads to higher adoption of legume-based rotations and to higher yields and gross margins. A study in Ethiopia [17] documents significantly higher yields for two ILVs. It has also been document that adoption of improved bean varieties has a positive impact on dietary diversity and reduces food insecurity, which they suspect is likely coming from the income effect resulting from the high yielding properties of these varieties [18].

This study carries out an analysis of plot-level, per unit area impacts of adoption of ILVs on yield and gross margins (defined in this paper as revenue minus cost of all inputs except land). By providing options for rotation, relay- and inter-cropping, legumes are vital components in diversification of Bangladesh’s predominantly rice-based cropping system and for enhancing yields of subsequent (for rotation and relay) and accompanying (for intercropped) cereals.

Plot-level evaluation of economic benefits of adoption of rotation, inter cropping, and relay cropping, especially associated with the introduction of ILVs, is best achieved by considering all benefits and costs from all crops during the entire cropping cycle [16]. While such an analysis is a subject of future research, due to data limitations, the analysis in this study is limited to only plot-level impacts on lentil yields and gross margins. Findings will be useful for researchers, policy makers, development practitioners, and extension personnel in Bangladesh and other countries in South Asia.

### Lentil production in Bangladesh

The agroclimatic conditions of Bangladesh are generally suitable for growing leguminous crops. Food legumes are traditionally cultivated under rainfed conditions, usually with minimum or no inputs other than seeds, land, and labor. Legumes are relatively short duration crops. Lentil is cultivated during winter (November-March), requires minimal tillage and fewer agricultural operations including weeding and no or minimal irrigation [19].

Due to their ability to fix atmospheric nitrogen (35–100 kg/ha/annum), lentils can play an important role in enhancing the sustainability of rice-based cropping systems [14]. In Bangladesh, lentils are grown in several ways—as a sole, mixed, or inter crop. While the most common cultivation pattern is sole cropping of short-duration varieties, sizeable area is also under intercropping with crops such as wheat, mustard, linseed, and sugarcane. Intercropping and mixed cropping are age-old practices, particularly in the North and North-western parts of Bangladesh [20]. The practice has developed as an informal insurance against a crop failure. Relay cropping in transplanted rice plots is common in low-elevation plots receiving water runoff from areas with higher elevation.

Between 1991 and 2015, BARI and BINA released 15 ILVs, developed individually or in collaboration with ICARDA (Table 1). Of these, in 2015, only eight were cultivated by farmers and these covered over 99% of national lentil area [8]. Due to their shorter duration, the introduction of the ILVs has made rice-based relay cropping more attractive. In this system, one crop is interplanted with a second crop as it approaches maturity. The practice is now common in rain-fed agro-ecosystems in Bangladesh. This system is suitable for post-monsoon cropping where a second crop in succession to rice is grown taking advantage of residual soil moisture after the rice harvest. The second crop is cultivated without tilling and with no application of fertilizers; seeds are broadcast about 2–3 weeks before the rice harvest [21]. Higher yields of rice were also found when grown after lentils [22]. Production of lentils also has the benefit of enhancing agrobiodiversity by breaking the pattern of rice mono-cropping and spreading labor and land use into previously idle periods.

**Table 1:** Lentil varieties released in Bangladesh between 1991 and 2015.

No.	Name of the variety	Year of release	Origin of germplasm	Selection History	Main traits (Characteristics)	Days to maturity	Yield from experimental stations (ton/ha)
1	BARI Masur 1 (ILL 5888)	1991	BARI/ICARDA	Selected from Pabna local (L15)	High yield and rust resistance; white flower color.	105–110	1.7–1.8
2	BARI Masur 2 (ILL 8007)	1993	BARI/ICARDA	ILX 113–55	High yield and rust resistance. Tendril present at leaf.	105–110	1.5–1.7
3	BARI Masur 3 (BLX 8405–56)	1996	BARI	Selection after Hybridization BLX 8405–56	High yield and rust resistance; Seed coat is greyish and spotted, seed size is bolder than local.	100–105	1.5–1.7
4	BARI Masur 4 (ILL 8006)	1996	BARI/ICARDA	ILX 87247	Resistance to Stemphylium blight (SB) and rust; high iron. High yield.	110–115	1.6–1.7
5	BARI Masur 5 (ILL 10847)	2006	BARI/ICARDA	X95-S136	Resistant to SB and rust; tolerant to foot rot; high yield.	110–115	1.4–1.6
6	BARI Masur 6 (ILL 10848)	2006	BARI/ICARDA	X95-S164(5)	Resistant to SB and rust; tolerant to foot rot; high in iron and zinc; high yield.	105–110	2.2–2.3
7	BARI Masur 7 (X95-S167(4))	2011	BARI/ICARDA	X95-S167(4)	Tolerance to SB and rust; red colour; High yield. Good cooking quality; high crude protein (30–31%)	110–115	1.8–2.3
8	BARI Masur 8 (LR 9–25)	2015	BARI/ICARDA	LR 9–25	Tolerance to SB and rust; micronutrient-dense variety (iron and zink); can be planted late; high yield.	110–115	1.8–2.0
9	BINA Musur 1	2001	BINA	Selection from Datura seed extract mutants	Seed coat colour is black, Grain reddish yellow, tolerant to SB	125–130	Max. 2.0 Av. 1.8
10	BINA Musur 2	2005	BINA	Mutant of Utfala	Early maturing, red colour with good cooking quality, higher protein (24–25%)	95–100	Max. 1.9 Av. 1.8
11	BINA Musur 3	2005	BINA	Mutation	Moderately resistant to rust, foot and root rot/wilt diseases, pod borer and tolerant to mild water stress, late sowing potential	95–100	Max. 2.4 Av. 1.8
12	BINA Musur 4	2009	BINA/ICARDA	Mutation	Moderately resistant to rust, foot and root rot/wilt diseases; a good cooking quality.	96–102	Av. 1.8
13	BINA Musur 5	2011	BINA/ICARDA	Mutant of BARImasur-4 with 200 Gy dose	Tolerant to blight and rust diseases, red colour with good cooking quality crude protein (29–30%)	99–104	Max 2.2 Av. 2.15
14	BINA musur 6	2011	BINA/ICARDA	Mutant of BARImasur-4 with 250 Gy dose	Tolerant to blight and rust diseases, red colour with good cooking quality, crude protein (30–31%),	105–110	Max. 2.0 Av. 1.95
15	BINA Musur 7	2013	BINA	Selection from ICARDA germplasm	High yielding and tolerant to SB and rust	110–112	2.2 to 2.4

## Materials and methods

This study has obtained an institutional review board (IRB) clearance from the ICARDA Research Ethics Committee (REC) (Ref No. 2021-REC-ECR-02, ORAL CONSENT).

### Methods

Some economic benefits of new varieties are from higher yields and/or lower costs, both of which contribute to lower per-unit cost of production. Statistical identification of the effect of an adopted crop variety using observational data is known to be a challenge because adoption is a choice, and the evaluation must consider multiple sources of confounding. To overcome this problem, we employ a multivariate analysis where the outcome *Y* (yield or gross margins per ha) is regressed on a dummy variable *T* (taking a value of 1 if the farmer plants ILVs and 0 otherwise) and other household, farm, and farmer characteristics *X* including inputs as follows:

Y=θ+αT+γX+ε (1)

In this regression, omitted variables (such as differences in land quality, farmer motivation, skills, IQ among others) can affect the adoption decision (T) and the outcome variable (*Y*)—causing correlation between the error term and *T*–violating one of the assumptions of Ordinary Least Squares (OLS) regression.

Here, an instrumental variables regression (IV) approach is used. IV methods are commonly used in studies when observational data are used [23–26]. With experimental data, the confounders can be controlled for through the experimental design but use of observational data requires special care. An IV approach requires instrument(s) which are correlated with the decision to adopt but uncorrelated with the unobserved factors influencing the outcome [27–29]. Suppose there is endogeneity between the treatment variable *T* and the outcome variable *Y* in Eq 1. Suppose also that *Z* is a matrix of exogenous covariates that include valid instruments for *X*. Then the IV model can be described by Eqs 1 and 2, with the latter known as the selection equation.


T=ΠZ+μ (2)

Where Π is a vector of coefficients, *ε* and *μ* are the error terms and there can be overlap in X and Z. The fact that *Cov*(*ε,μ*) = *σ_με_*≠0 means that OLS estimation will lead to biased estimates of the coefficient; *σ_με_* is a measure of the level of endogeneity between the treatment and outcome. Two-stage least squares (2SLS) is used to estimate Eqs 1 and 2 and recover *α*, the parameter of interest. In the first stage, due to the binary nature of the dependent variable in the selection equation and to account for possible causes of endogeneity between the treatment and outcome variables, the probit model is estimated first to generate the predicted probabilities as is the case with standard Stata commands such as *ivreg*. As the *ivreg* Stata command by default treats all independent variables in the selection equation as instruments, we have estimated the model in two steps using the predicted probabilities of adoption from the first step. While the parameter estimates in the second step are still unbiased, the standard errors are not valid and hence bootstrapping gives asymptotically valid approximations of the distribution of errors [30,31]. Therefore, we report coefficient estimates and bootstrapped standard errors.

A critical determinant of the quality of the analysis is the presence of a credible instrument. We use an indicator variable reflecting whether a farmer received government/project support for growing lentils at least one year before the survey was carried out in 2015 (support to grow lentil) as an instrument. Even if a farmer cultivates lentils in multiple years, support for growing lentils was provided only once and it was given in the form of one or more of low interest credit, free lentil seed (1–2 kg certified seed of lentils) and fertilizers (adequate to grow the seed). Many farmers in Bangladesh either don’t apply or apply low amounts of fertilizers to legume crops. Therefore, in the government support project, fertilizer is given to demonstrate to farmers that adding phosphorus fertilizers in legume production is beneficial. The support was made available for all farmers and hence there was no criteria to discriminate between farmers. Therefore, the support was randomly given to anyone who asked for it.

The choice of the instrument is justified on grounds that, though not exclusive, certified seeds predominantly include latest improved varieties. We argue that this support, because it was provided in years past, does not have direct effect on current outcomes—yield and gross margins per ha except through its effect on adoption. Whether endogeneity is a problem, and if so, the validity of the instrument are tested empirically in the results section. Selection bias can also be an issue because farmers may self-select in or out of the treatment (adoption of the improved lentil varieties) for which a decision has to be made whether to correct for it. However, the IV approach helps to control biases arising from self-selection [32,33], omitted variables [32] and due to inclusion of variables which we should not have controlled for [34]. IV method’s key advantage compared with other statistical methods used to analyze observational data is its ability to isolate exogenous effects of an intervention by excluding endogenous self-selection effects without having to directly measure such self-selection [35].

In order to be able to fit a variant of the Cobb-Douglas production function (including farmer characteristics as inputs) for yield, all continuous variables (such as yield, gross margins, lentil area, all quantities of inputs and age, education and experience of the household head) are logged. To overcome the problem of zero values, a constant of 1 was added to all values before the transformation. Several factors such as the amount of inputs applied are important in determining yield and, in turn, gross margins per ha. Therefore, all these were included as explanatory variables.

### Diagnosis of the choice and specification of the IV model

Before proceeding with estimation, diagnostic specification tests were conducted. First, we applied the Durbin [36] and Wu–Hausman [37,38] test statistics both of which rejected (at p<0.01) the null hypothesis that the treatment is exogenous–showing that endogeneity is a problem. Hence, the use of a model that corrects for endogeneity is necessary. Then, the correlation between the instrument and the endogenous variable (adoption) was examined. The two variables have a correlation of 0.16, significant at 0.01 level. The yield and gross margins equations were also estimated using OLS to generate residuals. The correlations between the residuals from the yield and gross margins equations and the support dummy variable (the instrument) were 0.04 and 0.05, respectively, both statistically insignificant showing that the instrument is valid. We also followed [39] and carried out a falsification test, which showed that the instrument had a positive and significant (p<0.01) effect on the adoption decision but no significant effect (p>0.1) on both per ha yield and gross margins of the non-adopters. Unless there is another variable in the equation which is highly correlated with the instrument which might cause the instrument to be insignificant (which is not the case in our regression), an insignificant coefficient on the instrument is an indication of the absence of endogeneity.

With a single instrument, there is no way to test the exclusion restriction. As a result, we are forced to assume that the model is identified. Even with two or more instruments, one can put some level of assurance by testing for overidentification restrictions, but these tests are all based on an assumption that the model itself is identified, which cannot be tested, because it involves the structural error term. Therefore, while we have eliminated the obvious cases of failures, we would like to caution the reader that our instrument can still fail.

As proposed by [40], the Stock and Yogo minimum eigenvalue statistic which is reported after the estimation of the Two Stage Least Squares Estimation (2SLS) was used to test if the instrument is weak. With an F-statistic value of 21.4 which is far greater than the critical values of the minimum eigenvalues, we reject the null hypothesis that the instrument is weak. After careful investigation of the diagnostic results, we conclude that our model is correctly specified, and the instrument is theoretically plausible, empirically valid, and strong enough to adequately identify the effects of adoption of ILVs on the outcome variables.

### Data

This study focuses on ten major lentil-growing districts in western Bangladesh, constituting about 74% of total national lentil area. A complete sampling frame listing all lentil growing farmers in all ten districts in western Bangladesh was not available, making use of simple random sampling infeasible. Elicitation of adoption estimates from a panel of experts showed that the ILVs were cultivated by over 80% of farmers in Western Bangladesh. Therefore, power analysis was then used to determine the minimum sample size (*MSS*) required to ensure at least 95% confidence and 2% precision levels for our estimates to capture between 20% and 80% adoption levels. The *MSS* was determined to be 864 but to compensate for possible non-response and missing data and to ensure adequate statistical power, we increased the sample size to 1,000.

The sample was drawn using a multi-stage stratified sampling technique. First, all ten districts were purposively included. Second, a random sample was drawn to select 20 sub-districts (locally called *Upazillas*) and 52 villages. The sample was distributed among the 20 *Upazillas* and 52 villages in proportion to the total number of lentil growers in each district, and sub district while the sample size per village was fixed between 17 and 19 (Table 2).

**Table 2:** Distribution of samples across Western Bangladesh.

District	*Upazilla*	Number of lentil farmers in the *Upazilla*	Lentil area in the *Upazilla* (ha)	Number of sample villages	Number of sample households
Chuadanga	Alomdanga	80090	817.41	3	58
Chuadanga	Chuadanga Sadar	44795	958.70	3	57
Faridpur	Faridpur sadar	42120	2884.62	3	54
Faridpur	Modhukhali	26500	2029.15	3	54
Jessore	Bagharpara	35605	1425.91	3	56
Jessore	Jessore Sadar	64282	977.73	3	59
Jhenaidah	Jhenaidah Sadar	58732	809.72	4	72
Jhenaidah	Kaliganj	40847	1251.01	4	71
Kushtia	Kumarkhali	38768	2024.29	2	41
Kushtia	khuksha	16808	1360.32	2	43
Magura	Magura Sadar	53129	1940.49	2	33
Magura	Sreepur	26749	2761.13	2	31
Natore	Bagatipara	20656	2202.43	2	48
Natore	Baraigram	43595	3528.34	2	45
Pabna	Chatmohor	39466	1111.34	2	36
Pabna	Ishardi	32378	2940.08	2	42
Rajbari	Pangsha	54116	1808.50	2	40
Rajbari	Rajbari Sadar	36741	1315.79	2	40
Rajshahi	Charghat	47896	848.18	3	61
Rajshahi	Puthia	32486	1062.35	3	59
**Total in the sample *Upazillas***		**835,759.00**	**34,057.49**	**52.00**	**1,000.00**

Note: Source of data on total lentil area is the Bangladesh Bureau of Statistics, 2011. Total number of lentil growers are generated through discussion with district level authorities. The percentage shares are authors’ own calculation.

An average of 20 households per village were randomly selected from a master file containing the list of all lentil growing households in the village. The questionnaire, enumerated to the person most knowledgeable about lentil cultivation, covered household demographic and economic conditions, asset ownership and other relevant factors. Information on lentil farming was obtained by asking detailed questions on varieties planted (farmer recall), input use, management practices, yields and factors of production for all lentil plots. Community-level information on access to infrastructure, farm services, extension, etc. was obtained from a separate village-level survey.

The variety was also verified using DNA fingerprinting, to ensure that farmer knowledge of the specific variety was not a source of error. The DNA fingerprinting process began with collection of reference seed samples where breeder seed samples were obtained for all released varieties from BARI and BINA. During the household survey, samples of seeds were collected from the 1694 plots planted by the 1000 sampled farmers. Prior to the analysis, a set of five Inter Simple Sequence Repeats (ISSR) and 41 Simple Sequence Repeats (SSR) markers [41,42] were identified from the lentil genome covering different linkage groups and individually tested for polymorphism in the breeder samples. Two ISSR and 20 SSR markers showed significant polymorphism across the Breeder seed samples and were used for varietal identification. For more details about the procedures used for DNA fingerprinting, we refer the reader to [8]. There was an 89% concordance between farmer recall and varietal identification using DNA-fingerprinting–showing reasonably accurate recall [8]. All analysis carried here is based on the DNA-verified varietal identification.

The survey was enumerated in 2015. During a stakeholders meeting, breeders from BARI and BINA, extension personnel from the district offices, and staff of the Bangladesh Agricultural Development Corporation (BADC), the main public seed producing and marketing entity, agreed that only varieties released after 2006 are considered improved. To exclude farmers who are testing the varieties for the first time and might eventually not adopt them, we agreed that adopters must be those who cultivated ILVs for at least 2 years. Therefore, for the purpose of this study, the definition of adoption is use of ILVs that are released after 2006 for at least two years.

About 48.8% of sampled household have adopted ILVs released in or after 2006. With an average of about 45 and 7 years, the age and education of the household heads in adopter and non-adopter groups are not statistically different while the farming experience of the household heads in the adopter households is statistically greater (at 0.01 level). Farmers who received support to grow lentil and those who purchased certified seeds are found to adopt the improved lentil varieties more than those who didn’t receive any support and those who didn’t buy certified seeds. Table 3 provides summary statistics for selected variables including those used in the IV model.

**Table 3:** Summary statistics for variables included in the models.

Variable name	Variable	Adopted improved lentil varieties—ILV (Adopter = 1)		Did not adopt improved lentil varieties—ILV (Adopter = 0)		Entire sample		
		Mean or count values	Std. dev.	Mean or count values	Std. dev.	N^	Mean value	Std. dev.
	**Variables derived from household-level data (N = 1000)**	***488***		***512***		**1000**		
Age	Age of household head (years)	45.55	11.38	46.25	11.67		45.91	0.36
Educ	Education of household head (years)	7.07	0.20	6.78	0.20		6.93	0.14
Exp	Farming experience of the household head	27.05***	0.49	23.80	0.48		25.39	0.34
Exec	Household head is an executive in the local administration (0 = No, 1 = Yes)	0.10	0.01	0.10	0.01		0.10	0.01
PVS	Involved in participatory variety selection (PVS) (0 = No, 1 = Yes)	0.24	0.02	0.01	0.02		0.01	0.01
Support-to-grow-lentil	Did the household receive support to grow lentils (0 = No, 1 = Yes)	0.18***	0.01	0.07	0.01		0.12	0.01
	**Variables derived from plot-level data (N = 1,694)**	***747***		***947***		**1,694**		
Seedkg	Quantity of seed used (kg/ha)	52.92***	0.57	56.22	0.57		54.76	0.42
Ureakg	Quantity of nitrogen fertilizer (Urea) used (kg/ha)	35.61	1.42	37.54	1.27		36.68	0.95
Dapkg	Quantity of DAP fertilizer used (kg/ha)	7.94	0.83	9.76	0.84		8.96	0.59
Tspkg	Quantity of Triple Super Phosphate (TSP) fertilizer used (kg/ha)	72.25	2.59	66.79	2.13		69.20	1.65
Mopkg	Quantity of Muriate of potash (MOP) fertilizer used (kg/ha)	42.22	1.31	39.84	1.12		40.89	0.85
Fungicidegm	Quantity of fungicides used (gm/ha)	244.75	14.13	257.49	13.38		251.87	9.73
Insecticideml	Quantity of insecticides used (ml/ha)	284.29***	14.65	332.48	13.60		311.23	9.99
vmech	Value of machinery inputs (Taka/ha)	1621.30	58.40	1630.00	46.08		1626.16	36.41
Totallabor_ha	Total labor spent for lentil production on this plot (days/ha)	46.93	0.80	46.08	0.66		46.45	0.51
Numirrig	Number of times the lentil plot is irrigated during the growing season	0.78	0.02	0.82	0.02		0.80	0.01
Lent_plot_Area_Ha	Size of lentil plot (ha)	0.21	0.01	0.20	0.01		0.20	0.01
Certified_seed	Was certified seed used on this plot (0 = No, 1 = Yes)	0.16***	0.01	0.07	0.01		0.11	0.01
Duration	Number of days between planting & harvesting of the lentil crop on this plot	151.05**	1.52	147.08	1.26		148.83	0.97
SWcons	Plot has soil and water conservation structures (0 = No, 1 = Yes)	0.03	0.01	0.03	0.01		0.03	0.01
Sole	Lentil was grown as a sole crop (0 = No, 1 = Yes); 0 = as a relay or intercrop	0.95***	0.01	0.92	0.01		0.93	0.23
Tworice	Lentil was grown between two rice crops (0 = No, 1 = Yes)	0.04***	0.01	0.08	0.01		0.06	0.24
DiseasePest	Plot was affected by disease and/or pests (0 = No, 1 = Yes)	0.49**	0.01	0.55	0.01		0.53	0.01
Kushtia	This field is located in Kushtia region (0 = No, 1 = Yes)	0.21***	0.01	0.14	0.01		0.17	0.01
Rajshahi	This field is located in Rajshahi region (0 = No, 1 = Yes)	0.19	0.01	0.22	0.01		0.20	0.01
Pabna	This field is located in Pabna region (0 = No, 1 = Yes)	0.08	0.01	0.07	0.01		0.08	0.01
Jessore	This field is located in Jessore region (0 = No, 1 = Yes)	0.31	0.02	0.33	0.01		0.32	0.01
Faridpur	This field is located in Faridpur region (0 = No, 1 = Yes)	0.18**	0.01	0.23	0.01		0.21	0.01
Yield	Lentil yield (kg/ha)	1444.41	17.63	1451.34	16.36		1448.28	12.00
Grossmargin	Gross margins (Taka/ha)^#^	90099.68***	700.99	75671.56	790.04		82033.90	566.36

^ N indicates the number of cases with a “Yes” answer and ***bold-italic*** figures represent count values.

^#^ Gross margin is defined as revenue minus cost of all inputs except land. The exchange rate in 2015 was 1US$ = 77.94 Bangladeshi Taka.

***, **, * represent significant difference between adopters and non-adopters of ILV at 0.01, 0.05 and 0.1 levels, respectively.

## Results and discussion

### Factors affecting adoption

Results of the first stage of the 2SLS estimation of the IV model are presented in Table 4. Parameter estimates of the selection equation (or first stage estimation) show that adoption of ILVs was positively and significantly (all at 0.01 level) affected by the education level and farming experience of a household head, the use of certified seed, whether a plot is located in Kushtia, and Pabna regions, and whether a farmer obtained support to grow lentil. These results are all consistent with theoretical expectations because educated and more experienced farmers are likely to better understand the opportunities and challenges involved in adopting a new variety. Moreover, 99% of certified seeds sold in western Bangladesh in 2015 were for ILVs and hence anyone buying certified seed was highly likely to also be using ILVs. Given that Faridpur (the province which was dropped from the equation) is predominantly irrigated, these lentil varieties which were mainly bred for rainfed areas may not fit well in the region for which farmers located in the relatively drier provinces of Kushtia and Pabna are highly likely to adopt the ILVs. As argued in the selection of the instrument, support for growing lentil is expected to motivate farmers to adopt improved varieties.

**Table 4:** Parameter estimates of the instrumental variables model using 2SLS method.

Independent variables^	Adoption of ILV (No = 0, Yes = 1)		Yield equation (lnyield)		Gross margin eq. (lngrossmargins)	
	Coef.	Std. Err.	Observed Coef.	Bootstrap Std. Err.	Observed Coef.	Bootstrap Std. Err.
ILV			1.34E-01	0.05**	0.16	0.04***
lnAge	-1.56	0.19***	0.21	0.11**	0.05	0.08
lnEdu	0.11	0.04***	-0.03	0.01***	-0.01	0.01
lnExp	1.07	0.15***	-0.13	0.07*	-0.02	0.06
Exec	0.23	0.10**	-0.09	0.04**	-0.02	0.03
PVS	0.03	0.53	0.29	0.07***	0.23	0.06***
Certified_seed	0.41	0.11***	-0.08	0.04**	-0.02	0.03
Tworice	-0.54	0.15***	0.10	0.05**	0.10	0.04***
Sole	0.31	0.15**	-0.10	0.05**	-0.07	0.03**
Kushtia	0.34	0.10***	0.16	0.04***	0.09	0.03***
Rajshahi	0.07	0.10	0.03	0.04	0.00	0.03
Pabna	0.44	0.15***	0.18	0.05***	0.13	0.04***
Jessore	0.09	0.09	0.16	0.03***	0.10	0.02***
Support-to-grow-lentil~	0.45	0.10***				
Lnseedkg			0.09	0.04***	0.06	0.03**
Lnureakg			0.00	0.01	0.00	0.00
Lntspkg			-0.02	0.01	0.00	0.01
Lnmopkg			0.01	0.01	0.01	0.01
Lndapkg			0.01	0.01	0.02	0.00***
Lnfungicidegm			0.01	0.00***	0.00	0.00*
Lninsecticideml			0.01	0.00*	0.01	0.00**
Lvmech			0.03	0.01***	0.00	0.01
Lntotallabor_ha			0.13	0.02***	0.09	0.01***
Lnnumirrig			0.07	0.03**	0.03	0.02*
Lnduration			0.10	0.04**	0.05	0.02*
DiseasePest			0.08	0.02***	0.03	0.01*
_cons	1.71	0.57***	5.23	0.4***	10.30	0.23***

***, **, * represent significant difference between adopters and non-adopters of improved lentil varieties at 0.01, 0.05 and 0.1 levels, respectively.

^ ln in front of all continuous explanatory variables indicates that natural logarithm of the variable is used in the regression.

~ The dummy variable support-to-grow-lentil (whether the farmer received support to grow lentils) is the instrument used for adoption of lentil varieties released after 2006 (ILV).

Age of household head and whether lentil is planted between two rice crops negatively and significantly affect adoption of ILVs. These results are also theoretically sound as older farmers are often overly attached to their age-old varieties. Plots on which a farmer plans to plant rice before and after the middle crop is less likely to be planted to lentils because jute, mung-bean, and sesame, which are summer crops, can be planted and harvested successfully in the spring/summer season. For a successful rice-lentil-rice system, lentil, which is a winter crop, has to be planted in November and harvested in February making it cumbersome for farmers. In the absence of super-early lentil varieties, farmers prefer to plant jute, mung-bean or sesame after lentil which can be harvested much earlier (by November) and get some rest for themselves in Dec-Jan. That is why area under lentils between two rice crops is much less than that which is under rice-lentils–jute/mung-bean/sesame. Improved lentil varieties called BARI_Masur 7 and 8 released by BARI and ICARDA after 2011 are said to be super-early and are expected to make farmers’ dream of the rice-lentil-rice cropping system a reality.

### Impacts of improved lentil varieties

This paper conducts plot-level impacts of varietal replacement (i.e., replacement of land races and old improved varieties) by most recent improved varieties. For the purpose of this study, adoption is defined as the use, for at-least two years, of lentil varieties that have been released since 2006. This means, the counterfactual group in this study comprises of mostly old, improved varieties which originated from BARI, BINA and ICARDA with only 16 cases of other varieties suspected to be landraces. Therefore, the impacts measured in this study represent the impacts of lentil varietal replacement in Bangladesh. These types of impacts are described by [43] as impacts of Type II technical change in post-green revolution agriculture where farmers regularly replace modern varieties to maintain disease resistance and to take advantage of the ever-higher yields of newer varieties.

#### Impacts on yields

Before presenting the impact of ILVs adoption at the plot level, it is important to discuss how different factors affect the yield of lentils in Bangladesh. 2SLS estimates of the outcome the outcome (yield) equation presented are columns 3 and 4 in Table 4. The results provide evidence that appropriate seed rates, fungicide, labor use, the cost of all machinery inputs, whether the plot had stress from disease and/or pests and whether plot is located in Pabna, Kushtia, and Jessore regions—all have positive and significant (at 0.01 level) effect on yield. Other inputs which have positive and significant effects are the number of irrigations (p<0.05) and application rates of insecticides (p0.1). Other factors having positive and significant effects on yield include farmer’s involvement in participatory variety selection (p<0.01) and the duration of stay of the lentil crop on the plot (p<0.05), farmer age (p<0.05).

The positive effects of the rates of the inputs listed above show that the Bangladeshi farmers are producing at apply quantities which are below the marginal product-maximizing levels. Long-held belief that legumes don’t need much care as well as problems related to financial liquidity among the predominantly poor farmers are responsible for the low input levels. The positive and significant effect of the duration of stay of the crop on the plot is also consistent with agronomic practices because, the longer growth period enables the crop to draw more nutrients and soil moisture, and hence produce more. The positive effect of diseases and/or pest prevalence is, however, counterintuitive. A positive correlation between pest prevalence and land quality and/or location of the farm within the regions (districts and *Upazillas*) might provide a possible explanation for the positive and significant coefficient on the pest prevalence variable.

To our surprise, several factors have negative effects on yield which include the household head’s education (p< 0.01) and membership in the Village Executive Committee (p< 0.02), the use of certified seed (p< 0.05) and whether lentil was grown by itself as a sole crop instead of inter/relay cropped (p< 0.05). Being a village executive might take the farmers’ time away from their plots—thereby causing them to compensate it with hired labor which is often not equally effective. When lentil is cropped in a relay or intercrop with other crops, it tends to take advantage of the residual moisture for its growth as opposed to when it is sown as a sole crop during the relatively dry season. The negative effects of the other variables appear to us counterintuitive. Education is meant to equip the farmer with the needed knowledge and understanding of the varieties and the associated agronomic practices. Likewise, theoretically, the use of certified seed is meant to enhance productivity. Given that lentils are self-pollinated, farmers are unlikely to purchase certified seeds every year. Therefore, farmers who purchase certified seeds are recent adopters and hence have short experience with the ILVs. This might make such farmers at a disadvantage in terms of yield.

After controlling all the confounding factors, the impact of adoption of ILV is found to be positive and significant (at 0.05 level). This result is in contrast with the bivariate comparison between yields of adopters and non-adopters shown in Table 2 which shows no significant difference. In a log-linear specification, the percentage change in the continues dependent variable in response to the change in a dummy variable from 0 to 1 (in our case from being a non-adopter to an adopter) is given by 100%*(*exp*(β)-1), where exp stands for the exponentiation operator. Therefore, the 0.134 coefficient estimate on the ILV dummy variable means that cultivation of an ILV leads to an average of 14.3% yield increase relative to cultivation of varieties released before 2006 and landraces together. Given the average yield of 1263.27 kg/ha that the adopters would have obtained had they not adopted, the 14.3% yield gain due to the adoption of ILVs translates to about 181.14 kg/ha. This level of yield gain is about half as much as the 27% (438 kg/ha) yield gain obtained from experimental plots (Table 5). For the experiments, the different lentil varieties were planted as sole crops under similar conditions including location, input use, preceding crop, planting and harvesting dates, biotic and abiotic stresses etc. Moreover, in our sample, we have 13 observations in the counterfactual group with extremely low average yield of 891kg/ha which are more likely to be local varieties. During the interview, the farmers who cultivated these varieties said they are local varieties and during DNA fingerprinting, none of them matched any of the 15 improved lentil varieties in the reference library–making it more likely for these varieties to be landraces or old improved varieties unofficially brought into Bangladesh from neighboring countries by farmers on the border.

**Table 5:** Yield gains from improved lentil varieties (ILVs) based on data from experimental stations.

Variety group	Name	Release year	Area share (%) from DNA fingerprinting	Rank of area share	Average yield from experimental stations (t/ha)	Area-weighted average yield (t/ha)	Yield gain from varietal replacement based on experimental data (%)
Varieties released before 2006 (Old improved varieties)	BARI Masur 1	1991	0.17	9	1.75	1.622	27%
	BARI Masur 2	1993	0.29	8	1.6		
	BARI Masur 3	1996	29.86	2	1.6		
	BARI Masur 4	1996	22.37	3	1.65		
Varieties released after 2006 (ILVs)	BARI Masur 5	2006	10.54	4	1.5	2.06	
	BARI Masur 6	2006	31.24	1	2.25		
	BARI Masur 7	2011	3.77	5	2.05		
	BINA Musur 5	2011	0.62	6	2.15		
	BINA Musur 6	2011	0.51	7	1.95		
**Total or average**			**99.37^**	** **	**1.83**	**1.83**	**0.44 t/ha**

Notes

^ The remaining 0.63% of lentil area is believed to be under local varieties (landraces).

In a study carried in Bangladesh, [44] documented 51% higher yields of the earliest 3 improved varieties (Bari-Masur 1, 2 and 3) over landraces. Therefore, even though our estimates are on the lower side, in the face of several confounding factors and the predominantly old, improved varieties in the sample which are in the counterfactual group, we are convinced that our results are reasonable.

For a robustness check, we also estimated the Ordinary Least Squares (OLS) regression, propensity score matching (PSM), an endogenous switching regression (ESR), and finally estimated a control function (CF) model. While OLS and PSM showed positive but insignificant yield effects, estimates of the ESR model showed that the estimate of yield impact from adoption was also positive (consistent with the IV estimate) but the magnitude of the impact was only 2%, which is much smaller than that of the IV (Table 6). This is expected because ESR, by default, includes all covariates in the outcome equation into the selection equation thereby creating confounding and specification errors. To overcome this issue, we followed [45] and estimated a CF model in which we excluded the exogenous variables from the structural equation that explain variation in the endogenous explanatory variables.

**Table 6:** Results from endogenous switching regression (ESR) showing average treatment effects of adoption of improved lentil varieties on yield, and gross margins.

Sub-sample Effects	Yield (kg/ha)			Gross margin (i.e., total revenue-total cost other than cost of land) (Taka/ha)^		
	Decision state		Treatment	Decision state		Treatment
	To adopt	To not adopt		To adopt	To not adopt	
Farm households that adopted	1337.68	1313.97	23.71***	87,966.38	64,035.95	23,930.43***
Farm households that did not adopt	3249.313	1346.769	1902.54***	86221.94	71606.41	14,615.53***
Heterogeneity effects						

*** indicates significant effect at 0.01 level.

^The exchange rate in 2015 was 1US$ = 77.94 Bangladeshi Taka.

The exogenous variation induced by excluded instrumental variables provides separate variation in the residuals (or generalized residuals) obtained from a reduced form, and these residuals serve as the control functions [45]. The results of the CF model estimated manually showed that the coefficient on the predicted error term is not significant countering the results of the Durbin and Wu-Hausman tests presented in the methods section. However, in terms of impact, the CF model estimated manually and using the *etregress* command in Stata showed yield impacts of 8.21% and 13.34%, respectively. While the differing significance and magnitudes of impacts from the various models are bothersome, they are expected to behave in that manner as models such as OLS and PSM are known to have limitations in handling biases from observable and/or unobservable factors. The results from all the models which are known to correct for biases from all sources (and hence are more credible) are consistent in terms of the significance of the impacts of adoption on yield and gross margins but differ in magnitude due to the underlying assumptions. Therefore, we can conclude that adoption of improved lentil varieties leads to gains in yield and gross margins.

During the survey, farmers were asked about the traits they liked about the different improved lentil varieties and higher yield was important. For example, for BARI-6, out of 367 farmers who knew the variety, 96% said that they like the variety because it gives high yield, which is consistent with our findings. We also estimated a conditional logit model to identify variety-specific traits that influence farmers’ adoption decisions. Results show that disease resistance has the highest marginal utility to the farmers followed by yield and market prices (Table 7).

**Table 7:** Results of the maximum likelihood estimation of the conditional (fixed-effects) logistic regression model.

	Coefficient	Std. err.
Post2006	0.44	0.34
Yield	1.37	0.35***
Market price	1.10	0.24***
Taste	0.66	1.77
Disease_resistance	2.12	0.90**
Early_maturing	17.37	788.68

Notes

The dependent variable is adoption (as verified by DNA-fingerprinting) which takes a value of 1 if a farmer adopted improved varieties and 0 otherwise.

**, and *** represent significance at 0.05 and 0.01 levels, respectively.

As discussed in the introduction section, disease outbreak was the main reason for the introduction of the improved lentil varieties in the early 1990s and our results confirmed that this trait is the single most important even for newer varieties. Yield and gross margin advantages are also often associated with most technology adoption decisions including lentils in Bangladesh making our results consistent with the findings of other studies [15,16]. Despite the short season between two rice crops, duration does not seem to be important in varietal choice. Likewise, taste is not found to have a significant effect on farmers’ lentil variety choice. The results also revealed that farmers are indifferent to the age of the varieties, implying that farmers are not necessarily resistant to the adoption of newly released varieties provided that it possesses the most preferred traits. Bivariate analysis of the variation of yield using different variables (including farm size, age, experience, education, etc.) showed that there is no systematic difference in yield.

At the adoption level of 45% of the total lentil area of 107,549 ha in Bangladesh estimated by [8], the introduction of ILVs have increased total lentil supply from domestic sources by 8,259 tons (5%). Considering the average price of US$865/ton that Bangladesh paid for lentil imports in 2015, the introduction of ILVs in Western Bangladesh had saved the country hard currency of about US$7.14 million.

#### Impacts on gross margins

Due to the lack of a suitable variable in our data and the difficulty to find a good measure of the value of land, especially for owned lands, we decided to exclude it from the costing of lentil production. Therefore, in this study, gross margins (GM) is defined as the difference between revenue and the sum of all input costs except the cost of land per ha. The parameter estimates of the IV model for GM are also provided in columns 6 and 7 in Table 4. The GM equation contains the same variables as the yield equation. With few exceptions, the sign and significance of most of the variables are similar to that of the yield equation which should not come by surprise. This is because, yield is one of the major determinants of GM and hence, unless some of the inputs had prices which are higher than the value of their marginal product, we should expect the sign and significance of the estimates in both equations to be similar. Therefore, detailed discussion of the effects of each variable, especially those consistent ones is omitted here. It is however important to discuss the exceptions. For example, whether a farmer is an executive in the village and whether the farmer used certified seed do not have significant effects on GM.

The coefficient estimate on the ILV dummy variable in the IV model for gross margin is positive and significant. The coefficient estimate is 0.159 which, given that gross margins is in natural logarithms, means that adoption of ILVs leads to a 17.23% (13,244.98 Taka or US$169.94 per ha) increase in gross margins. The increase in gross margins is 2.89 percentage points higher than that of yield showing that adoption of ILVs does not only have yield benefits but also helps farmers in reducing costs of production and/or in fetching higher prices. This is supported by the saving on the quantities of inputs used by adopters of ILVs. For example, the amounts of seed and insecticides used by adopters are significantly lower than that of non-adopters (Table 2). OLS, PSM, and ESR models estimated for robustness check also showed gross margin gains of 19%, 1.9%, and 37%, respectively (ESR model results in Table 6)–showing that despite the differences in magnitude, adoption of improved lentil varieties indeed leads to higher gross margins.

## Conclusions

In Bangladesh, 15 improved lentil varieties (ILVs) developed by BARI and BINA individually and/or jointly with ICARDA were released between 1991 and 2015. This paper estimates plot-level impacts of adoption of these ILVs, also known as the impacts of variety replacement, i.e., the impact of replacing old, improved varieties (OIV) by recent improved varieties. We defined recent improved varieties as lentil varieties that were released in or after 2006 while the counterfactuals are predominantly OIV and other varieties (only 13 cases out of 1694 observations) which are suspected to be landraces.

Using an instrumental variables method, the adoption of ILVs is estimated to have average effects of 181.14 kg/ha (14.3%) higher yield and US$169.44/ha (17.23%) higher gross margins for the farmer. At the 45% adoption level estimated by [8], the introduction of ILVs in Bangladesh has increased total national supply of lentils by 8.77 million tons (6%) which reduced the imports by the same amount saving the country hard currency of about US$8.22 million annually. Our findings clearly show that Bangladesh has been reaping substantial benefits from the introduction of the improved lentil varieties of BARI, BINA and ICARDA origin. Despite almost complete replacement of landraces with improved lentil varieties, the adoption level of varieties released after 2006 (ILVs) is still low (45%). BARI, BINA and ICARDA have released more super early varieties after 2011 which are said to be even more advantageous with higher density of micronutrients through biofortification. The policy implication of our findings is that Bangladesh is not tapping into the full potential of the ILVs (and this might equally apply to similar countries in the South Asian region) and hence if these countries invest in efforts to disseminate them further, they have yet even greater benefits to reap.
