# Correction: Plot-level impacts of improved lentil varieties in Bangladesh

**DOI:** 10.1371/journal.pone.0335667

**Published:** 2025-10-29

**Authors:** Yigezu Atnafe Yigezu, M. Wakilur Rahman, Tamer El-Shater, Arega D. Alene, Ashutosh Sarker, Shiv Kumar, Aymen Frija

In the Abstract section, there is an error in the fifth sentence. The correct sentence is: Since 45% of lentil area is under ILVs, they generated over 8,766 tones (6%) more supply of lentils from domestic sources, saving the country US$7.58 million in imports in 2015 alone.

In the Impact on yields subsection of the Results and Discussion section, there is an error in the second sentence of the tenth paragraph. The correct sentence is: Considering the average price of US$865/ton that Bangladesh paid for lentil imports in 2015, the introduction of ILVs in Western Bangladesh had saved the country hard currency of about US$7.58 million.

In the Conclusions section, there is an error in the second sentence of the second paragraph. The correct sentence is: At the 45% adoption level estimated by [8], the introduction of ILVs in Bangladesh has increased total national supply of lentils by 8,766 tons (6%) which reduced the imports by the same amount saving the country hard currency of about US$7.58 million annually.
